# Systematic detection and evaluation of cracking behavior of flawed brittle sandstones with AE and 3D-DIC techniques

**DOI:** 10.1371/journal.pone.0309381

**Published:** 2024-09-06

**Authors:** Jie Fan, Xing Zhu

**Affiliations:** 1 School of Civil Engineering, Southwest Jiaotong University, Chengdu, China; 2 State Key Laboratory of Geohazard Prevention and Geoenvironment Protection, Chengdu University of Technology, Chengdu, China; 3 College of Computer Science and Cyber Security, Chengdu University of Technology, Chengdu, China; Shenyang Jianzhu University, CHINA

## Abstract

Determination of the cracking behavior during crack propagation helps to better understand damage and fracture processes in brittle rocks. The paper studies the cracking behavior of rocks on three scales: macro-deformation (or macro-cracking), internal micro-fracture, and surface crack coalescence. Under uniaxial compression, the cracking behavior of two types of sandstone specimens having single flaws was experimentally and systematically investigated. Acoustic emission (AE) and three-dimensional digital image correlation (3D-DIC) techniques were utilized to continuously monitor the acoustic shock signals generated by micro-fracture events inside the specimen and the specimen surface cracking process. The experimental results show that at the crack initiation stage, many micro-tensile fractures within the rock are initiated and coalesced, and small strain localized zones (SLZs) appear on the specimen surface. In the crack propagation stage, micro-fractures coalesce into macro-fractures that propagate in tensile mode to form surface cracks, which finally break in tension or slide against each other in shear mode. The formation of SLZs is related to the dip angle of pre-existing flaws, which determines the direction and mode of crack propagation. In conclusion, the strong acoustic-optical evidence accompanying different cracking behaviors is discussed in detail. From both acoustic and optical perspectives, it reveals and explains how flaws and material properties affect the strength and cracking mechanisms of brittle rocks. The study aids comprehension of the potential relation between internal micro-fracture and surface cracking in the process of engineering rock mass failure.

## 1. Introduction

As an important engineering medium, rocks develop initial flaws at various scales, such as pores and fissures, due to long-term geological function and weathering [1, 2]. These initial flaws of different shapes, sizes, and geometric distribution govern the expansion pattern of microcracks, causing the rock to exhibit distinct mechanical and deformation characteristics [3–5]. In addition, the mechanical characteristics of the rock mass are important parameters for rock engineering design and stability calculations. Therefore, understanding the cracking behavior of rocks during damage catastrophes is of great engineering importance and is one of the key challenges in rock fracture mechanics [6].

In the past decades, many methods have been proposed for detecting the cracking behavior of rocks based on physical test data or numerical simulations. Cracking behavior is a systematic and ambiguous concept and can be summarized in previous studies as macro-deformation [7, 8], internal micro-fracture [9] and surface cracking [10]. Macro-deformation can be regarded as the cracking of the entire rock and is commonly characterized in terms of volume strain. Bieniawski [8] classified the crack development process in compressed rocks into five stages: (1) Pre-existing microcrack closure. (2) The linear elastic deformation stage, where no cracks emerge. (3) Crack initiation and stable propagation. (4) Unstable crack propagation and coalescence. (5) Crack coalescence and penetration, complete failure of the rock. Each of the two adjacent stages is separated by a characteristic stress value corresponding to the crack closure stress *σ*_*cc*_, the crack initiation stress *σ*_*ci*_, the crack damage stress *σ*_*cd*_, and the peak stress *σ*_*p*_, respectively. Typically, researchers use strain data obtained by resistance strain gauges or extensometers to detect characteristic stress values in specimens [7, 11–13]. In recent years, with the rise of acoustic emission (AE) techniques, acoustic signal monitoring has also been widely used for the identification of stress thresholds associated with crack propagation [4, 14–17].

Internal micro-fracture of rocks refers to the micro-particle extrusion damage caused by the increased viscosity and friction between mineral particles and cement under load [18]. Therefore, identifying the patterns and occurrence mechanisms of internal micro-fracture events can provide a fundamental understanding of the cracking and mechanical behavior of rocks. Due to the mutual compression of mineral particles, micro-tensile failure and micro-shear failure coexist when micro-fractures occur. Thus, if the dominant mode in the damage process can be determined, reasonable designs and materials can be employed to strengthen the structure [9]. The internal damage and structural deformation of rocks are released by the accumulated elastic energy in the form of elastic waves or acoustic waves, i.e., the AE phenomenon. Therefore, the AE technique is one of the most common means to detect micro-fracture events inside rocks among previous studies [18–20]. AE characteristic parameters can also be employed to identify micro-fracture patterns in rocks, most commonly as a ratio of *RA* values (average frequency) to *AF* values (rise time/amplitude) for the characterization of micro-fracture mechanisms. In general, tensile fracture has a high *AF* value and a low *RA* value, whereas shear fracture has a high *RA* value and a low *AF* value [21–25]. In other words, determining the dominant fracture modes in rocks can play a crucial role in ensuring the stability and safety of rock engineering.

Unlike micro-fracture events within rocks, surface macroscopic cracks are the most intuitive manifestation of the evolution of rock damage. They are the result of the final stage of internal micro-fractures or separations. Rocks are discontinuous, heterogeneous, and brittle [1, 26–29], and their complicated internal structure creates uncertainty in crack initiation, development, propagation, and coalescence [30–32]. Therefore, the statistical surface crack propagation pattern can aid in our comprehension of the nonlinear degradation characteristics of rock’s strength and stiffness. In addition, there are several research findings about the propagation of cracks in similar materials [10, 33], crack monitoring [34, 35], pre-existing flaws [4, 36], and slope models [37, 38]. Cracking diminishes the mechanical properties of rock materials and is a prelude to fracture failure. Identifying the characteristics of crack initiation and propagation makes it feasible to predict the deformation behavior of rocks. As an intuitive and efficient monitoring tool, DIC is currently one of the mainstream monitoring techniques in rock crack propagation studies [39–43]. Commonly, surface cracks are divided into three types: tensile cracks, shear cracks, and hybrid cracks [44]. Previous studies have shown that tensile cracks are the most prevalent form of cracks during rock degradation, whereas shear cracks are typically directly related to the failure of the rock [9, 45, 46].

It is worth noting that tensile cracks and shear cracks in surface cracks and tensile fractures and shear fractures in internal micro-fracture events are distinct concepts that can easily be confused. After the elastic deformation stage, the emergence of surface fractures implies irreversible damage evolution of the rock [18]. In contrast, micro-fracture events are accompanied by friction between particles and exist throughout the loading process. Macro-deformation, internal micro-fracture, and surface cracking are independent and connected. Currently, there is a large amount of research on the crack propagation process in rocks with pre-existing single flaws [47–49], but few studies on the acoustic-optical correlation between different cracking behaviors have been reported. Existence of the following problems: (1) Reveal the potential relation and evolutionary mechanism among the macro-deformation, internal micro-fracture modes and surface crack types of rocks. (2) The correlation between strain information before surface crack occurrence and internal micro-fractures. (3) Acoustic-optical evidence of cracking-induced. (4) The relation between cracking behavior and initial flaws design. Understanding the physical processes of damage and fracture in brittle rocks will be enhanced by the resolution of the above issues.

To gain insight into the above problems, two types of brittle sandstone specimens were selected for uniaxial compression tests in this study. To boost the diversity of cracking behavior of the specimens, initial flaws with varying dip angles were created. The present study employs the AE technique to capture the acoustic emission characteristics inside the specimen and the 3D-DIC technique to monitor the full-field continuous deformation of the specimen surface. AE is used to measure the energy released during micro-fracture formation, and DIC measures the strain localized information of the specimen before surface cracks form. Based on the acoustic-optical-mechanical properties accompanying the rock damage process, the cracking behavior of brittle rocks, especially micro-fracture and surface cracks, is evaluated comprehensively.

## 2. Experimental design

### 2.1 Specimen preparation

As test materials for this study, two types of uniform and compact sandstones were utilized. The brownish-yellow sandstone with fine grains is referred to in this study as “fine yellow sandstone” for the sake of explanation. The second has a granular clastic structure with a dark red color and is referred to as “red sandstone”. Table 1 displays the average physical and mechanical parameters of the three intact sandstone specimens. Where *σ* is for uniaxial compressive strength, *E* is Young’s modulus, and *v* is Poisson’s ratio. Both fine yellow and red sandstone are brittle and hard.

**Table 1:** Physical and mechanical parameters of two types of sandstones.

Rock types	*σ*(MPa)	*E*(GPa)	*v*
Yellow Sandstone	49.6	6.8	0.21
Red Sandstone	27.22	6.9	0.26

**Fig 1:**
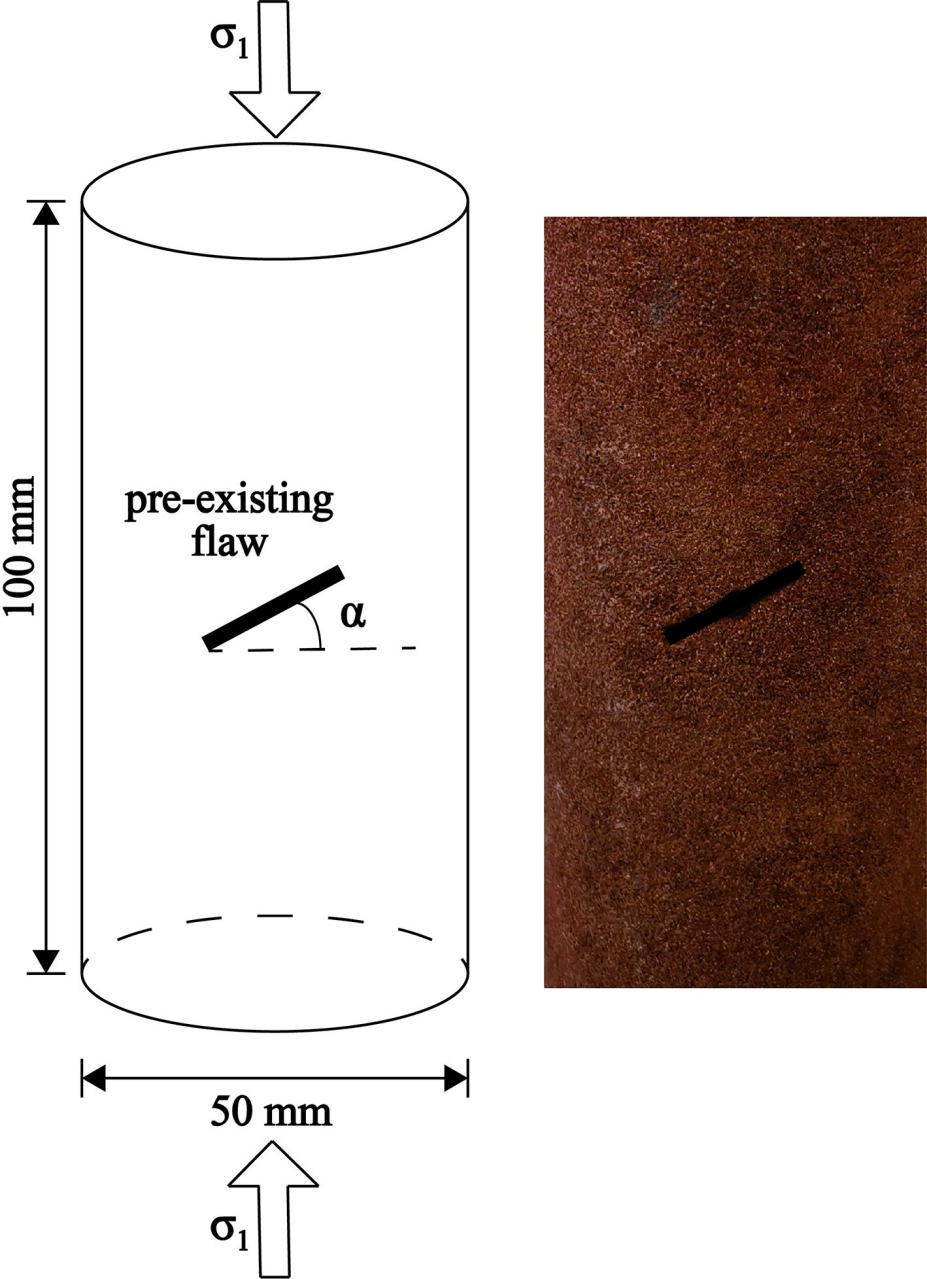
The geometry of the specimen and pre-existing flaw.

As depicted in Fig 1, the rocks were processed into standard cylindrical specimens of 50 mm in diameter and 100 mm in height by the ISRM recommended method [50]. The failure of intact rock specimens under uniaxial compression manifests as a single form of longitudinal tensile fracture. To enrich the cracking behavior of the specimens, single "flaws" with dip angles α ranging from 0° to 90° (15° interval for fine yellow sandstone and 30° interval for red sandstone) were prefabricated using the cutting technique, and the length and width of the flaws were 15 mm and 1 mm, respectively. There are three identical flawed specimens for each dip angle, and a total of 24 specimens of fine yellow sandstone and 15 specimens of red sandstone were tested in this study.

### 2.2 Experimental equipment setup

**Fig 2:**
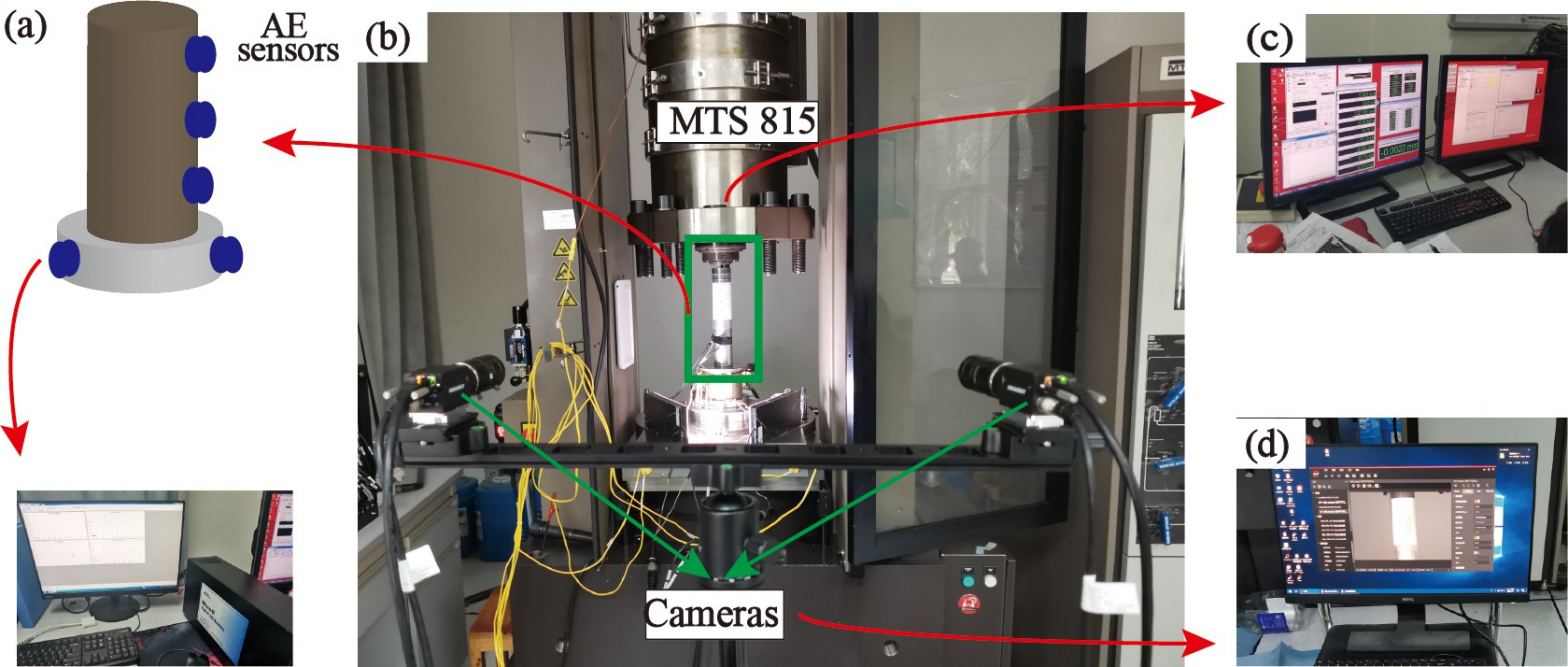
Experimental apparatus (a) AE desktop and the locations of the AE sensors, (b) photo of the test setup, (c) Mechanics acquisition desktop, (d) Image acquisition desktop.

On the MTS815 electro-hydraulic servo rigidity tester, uniaxial compression testing was performed. The servo valve of the tester is so responsive and accurate that it can meet the requirements of DIC deformation measurement. The acoustic signal monitoring system was the Micro-II AE system from Physical Acoustics Corporation (PAC), USA. The sampling frequency was set to 10 MHz. To reduce background noise, the sampling threshold is set to 45dB and the preamplifier gain is set to 40dB [51]. Since the front of the specimen requires image acquisition, three AE sensors are placed on the back of the specimen. The remaining three AE sensors are arranged in a ring shape on the force transmission pad in close contact with the rock specimen, as indicated by the blue dots in Fig 2(A). To ensure the integrity of AE signal propagation, petroleum jelly was applied between the sensor and the specimen, as well as between the specimen and the mat to improve the coupling effect. The equipment layout of the test is depicted in Fig 2(B), and the front side of the specimen is photographed by two industrial cameras on the left and right. Fig 2(C) shows the desktop for mechanical data acquisition.

**Fig 3:**
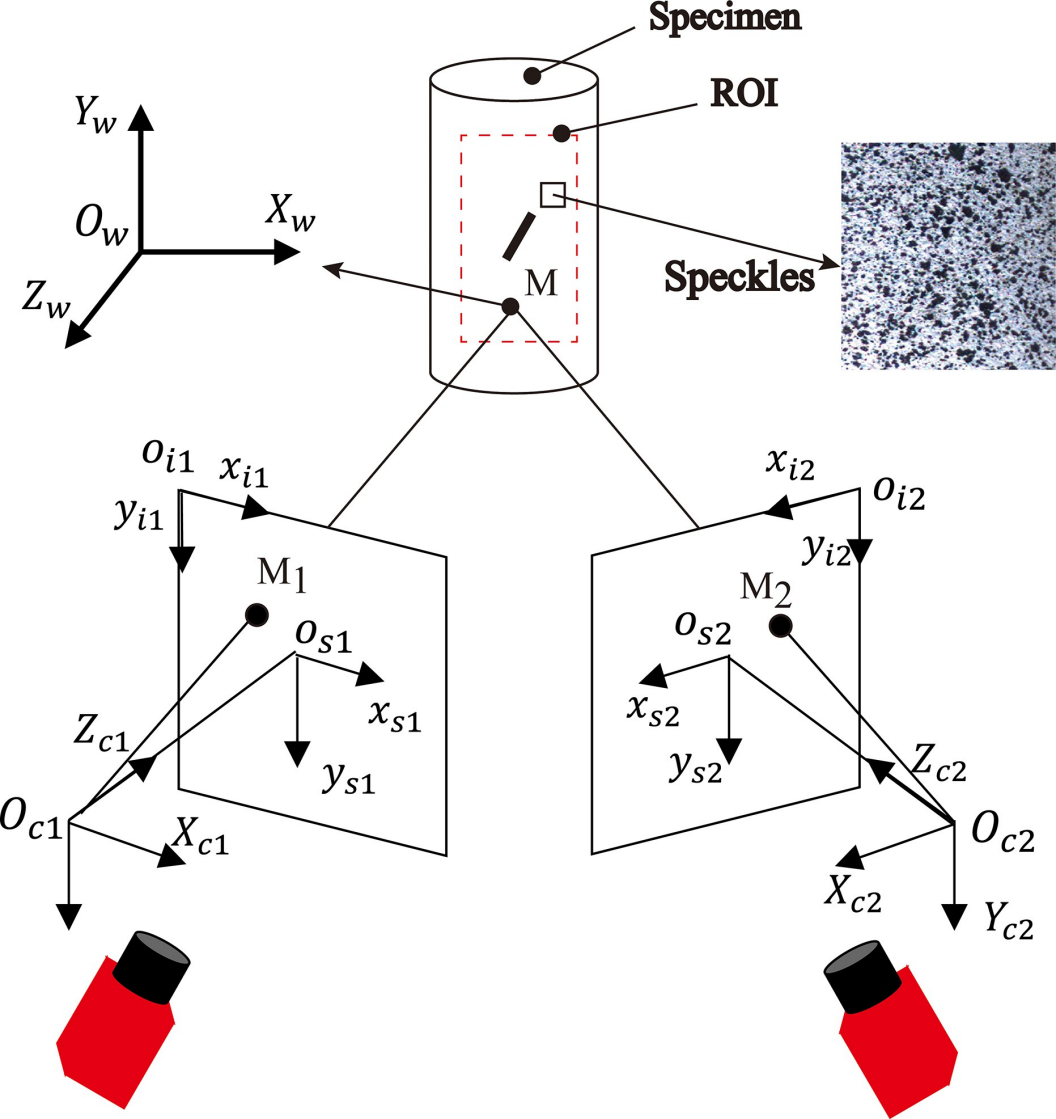
Schematic of stereo imaging setup [52].

The 3D-DIC system mainly consists of a hardware system responsible for image acquisition and a software system responsible for acquisition control and image processing analysis. The hardware system mainly includes two industrial cameras, light sources, camera mounts, calibration devices, scatter-making tools, etc. As illustrated in Fig 2(D), the software system comprises the image acquisition control system, the calibration system, the post-processing system, etc. The Multidic open-source software developed by Solav et al. [51] was utilized for the post-processing analysis of the scattered digital images in the present study. The basic principle of 3D-DIC is depicted in Fig 3 [52]. Based on the principle of binocular stereo vision and the image scatter matching technique, two cameras capture the region of interest (ROI) from different locations and reconstruct and track the 3D position of the ROI over time by stereo triangulation [51].

Before image acquisition, the acquisition system needs to be calibrated to obtain the internal parameters (geometric and optical characteristics) and external parameters (position and orientation with respect to the reference coordinate system) of the camera. Camera imaging involves four coordinate systems: the world coordinate system *O*_*w*_, the camera coordinate system *O*_*c*_, the image physical coordinate system *O*_*s*_, and the image pixel coordinate system *O*_*i*_, with the world coordinate system serving as the reference coordinate system. The reference coordinate system *O*_*w*_ is first transformed into the camera coordinate system *O*_*c*_ by means of external camera parameters, Secondly, the camera coordinate system *O*_*c*_ is converted to the image physical coordinate system *O*_*s*_. Finally, the image physical coordinate system *O*_*s*_ is converted to the image pixel coordinate system *O*_*i*_ by combining the internal camera parameters [51, 53, 54].

## 3. Experimental results

### 3.1 Macro-deformation levels

The process of deformation and damage to rocks results in an energy conversion. Thus, the macro-deformation level of the rock is determined by analyzing the cracking behavior of the specimen from the standpoint of strain energy (mechanics). According to the first law of thermodynamics [55], assuming that there is no heat exchange between the test system and the outsider, the dissipated energy generated by the internal damage and deformation of the rock under the affection of outside forces is calculated as follows [56].


$$U_{d}=U-U_{e}=\int_{0}^{\varepsilon }\sigma d\varepsilon -\frac{\sigma^{2}}{20E}$$ (1)

Where *U* (KJ/m^3^) is the total energy absorbed by the rock and *U*_*e*_ is the elastic strain energy that can be released. The axial stress σ (MPa) and axial strain ε (%) are calculated from Eq (2):

$$\left\{ \begin{array}{l} \sigma =\frac{P}{A}\\ \varepsilon =\frac{\Delta h}{h}\times 100\% \end{array}\right.$$ (2)

Where *P* is the axial load, *A* is the area of the loading surface, Δ*h* is the axial deformation, and *h* is the height of the specimen.

**Fig 4:**
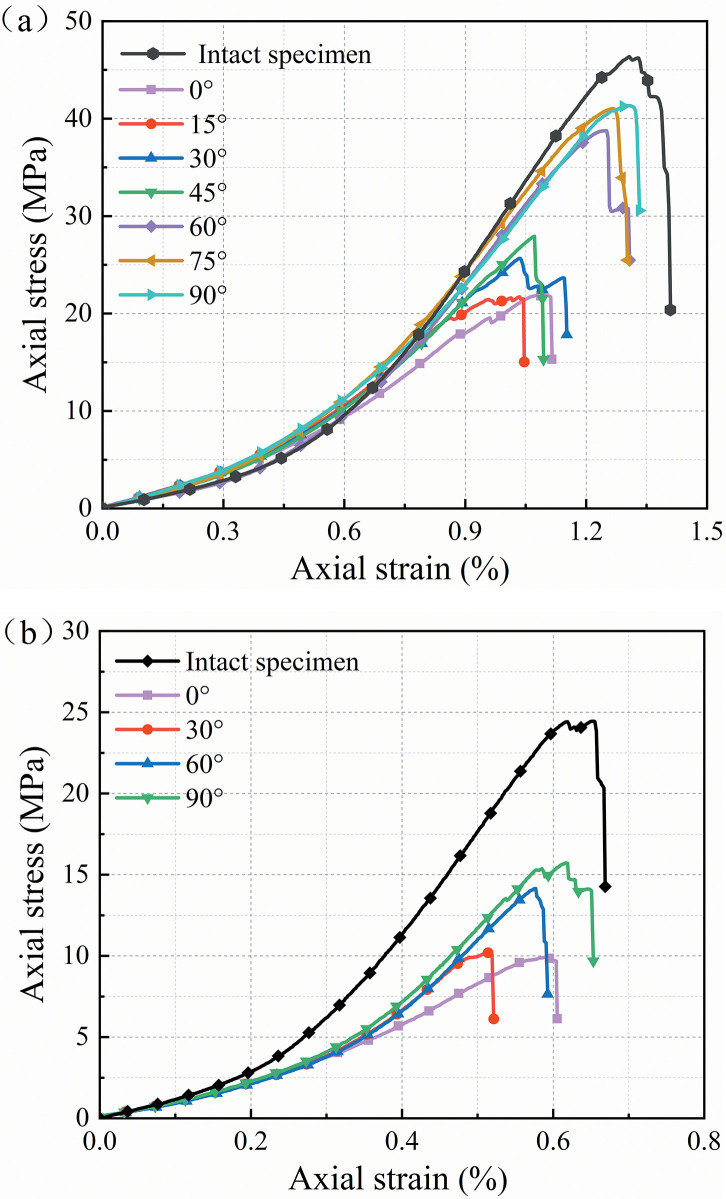
Stress-strain curves of different specimens (a) Fine yellow sandstone, (b) Red sandstone.

Fig 4 depicts the typical axial stress-strain curves of two sandstone specimen types during uniaxial compression. Since the mechanical properties of the specimens from the repeated tests were comparable, the data set with the better digital image acquisition was selected for analysis. As illustrated in Fig 4(A), the pre-existing flaws reduce the mechanical properties of the rock specimens. The peak stresses and peak strains of the fine yellow sandstone specimens containing single flaws range from 21.72 MPa to 41.35 MPa and 1.05% to 1.33%, respectively. When the angle of the flaw is 90°, the strength of the specimen is close to the peak stress of the intact rock specimen. When the angle is 0°, the strength of the specimen is approximately half of the peak stress of the intact rock specimen. The peak stress of the specimen containing a single flaw varies mainly in the range of angles between 45° and 60°. Initial flaws at lower or greater dip angle ranges have a lesser impact on the change in strength of rock specimens. The stress-strain curve variation pattern of the red sandstone is similar to that of the fine yellow sandstone, as shown in Fig 4(B). The red sandstone specimen has half the strength and deformability of the fine yellow sandstone specimen. As the inclination of the flaws rises, the stress-strain curve of the specimen becomes smoother before the peak stress and steeper after the peak stress.

**Fig 5:**
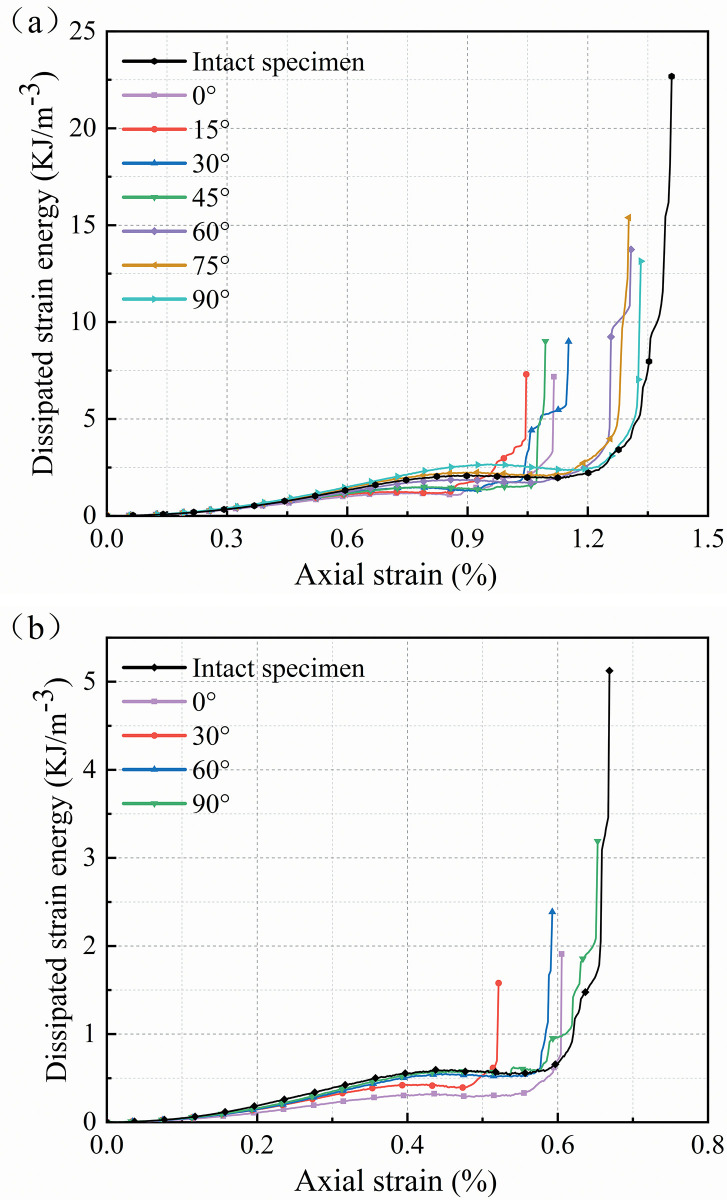
Evolution of the dissipated strain energy (a) Fine yellow sandstone, (b) Red sandstone.

Fig 5 shows the dissipative strain energy curves of the sandstone specimens. The evolution of the dissipative strain energy corresponds well to the rock damage progression. In the microcrack closure stage, the dissipation strain energy curve climbs gradually and remains flat in the elastic deformation stage. It implies that the energy dissipation during the early stage of loading is mostly due to the compression of the microcrack. In the microcrack closure stage, the nonlinear behavior of the strain energy dissipation curves of the specimens is comparable, but it appears discrete in the elastic deformation stage. The dip angle of the flaws appears to have little effect on the microcrack closure stage, but it does affect the elastic modulus of the sandstone, resulting in differences in the brittle characteristics of the sandstone. After crack initiation, the release of strain energy starts to increase. There are variations in the rate of rise of the dissipation energy curve as a result of differences in brittleness. Compared to the fine yellow sandstone, the red sandstone specimen in Fig 5(B) exhibits more serious brittle damage, and the strain energy is only released dramatically near the specimen failure. Table 2 divides the macro-deformation process of the specimen during a single test into four successive stages (cracking levels) for subsequent description. The maximum values of axial stress and strain for each stage are listed, which correspond to the crack closure stress *σ*_*cc*_, the crack initiation stress *σ*_*ci*_, and the damage stress *σ*_*cd*_, respectively [8]. The cracking behavior of brittle sandstone mainly occurs in stage III and stage IV.

**Table 2:** Macro-deformation stages of two flawed rock types.

Specimens	Angle (°)	Crack closure(Ⅰ)		Linear elastic deformation(Ⅱ)		Stable crack growth(Ⅲ)		Unstable crack growth and failure(Ⅳ)	
		Axial strain (%)	Axial stress (MPa)	Axial strain (%)	Axial stress (MPa)	Axial strain (%)	Axial stress (MPa)	Axial strain (%)	Axial stress (MPa)
Fine yellow sandstone	0°	0.35	4.25	0.71	12.26	0.88	17.79	0.88-	17.79-
	15°	0.26	3.32	0.66	12.46	0.81	17.91	0.81-	17.91-
	30°	0.33	4.22	0.65	11.73	0.85	19.40	0.85-	19.40-
	45°	0.50	7.59	0.76	15.72	0.89	21.12	0.89-	21.12-
	60°	0.64	11.19	0.98	27.49	1.05	31.11	1.05-	31.11-
	75°	0.70	14.79	1.03	31.23	1.15	37.74	1.15-	37.74-
	90°	0.96	25.86	1.12	34.43	1.21	38.96	1.21-	38.96-
Red sandstone	0°	0.24	2.77	0.39	5.46	0.46	7.13	0.46-	7.13-
	30°	0.18	1.88	0.35	5.06	0.40	6.80	0.40-	6.80-
	60°	0.14	1.35	0.35	4.96	0.44	8.05	0.44-	8.05-
	90°	0.16	1.70	0.37	6.06	0.46	9.75	0.46-	9.75-

### 3.2 Internal micro-fracture modes

**Fig 6:**
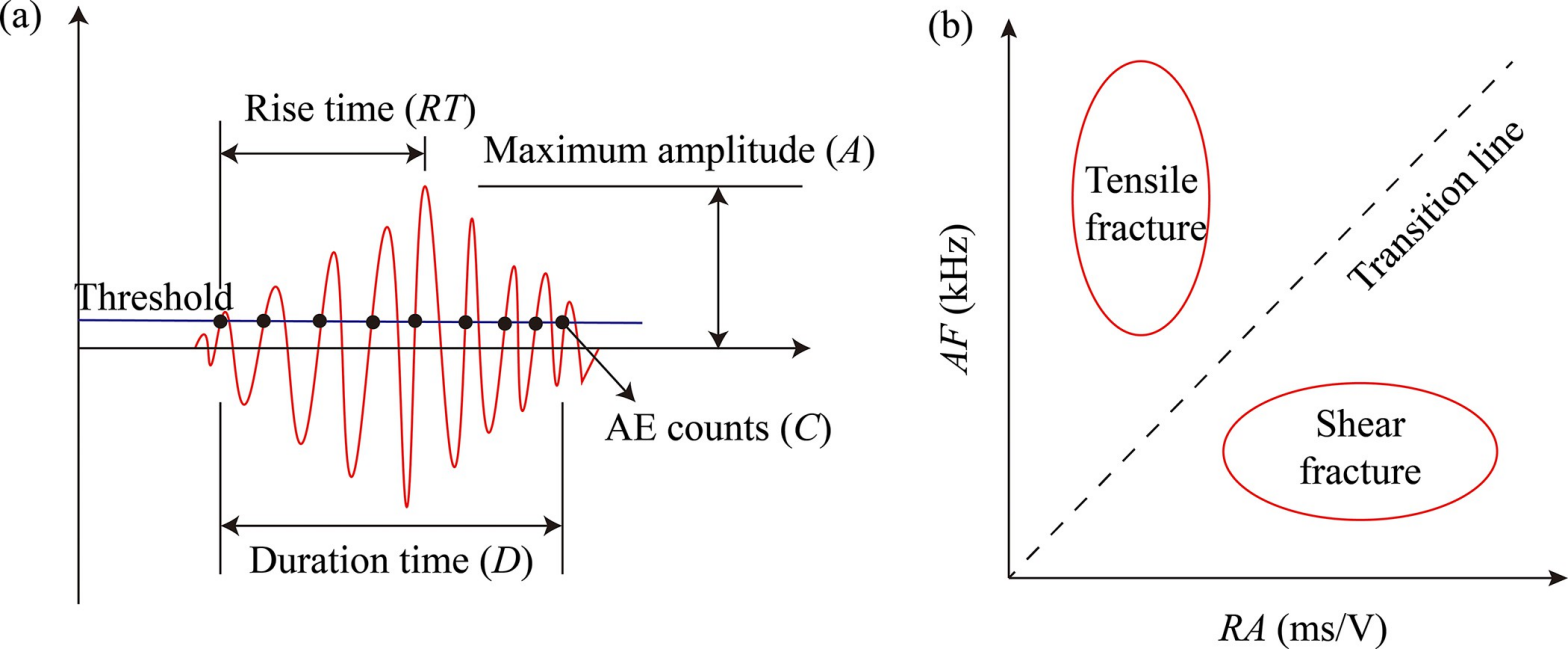
AE for detecting micro-fracture modes in rock materials (a) Definition of AE parameters, (b) Relation between the *RA* value and *AF* value [9].

As shown in Fig 6(A), different fracture mechanisms cause different types of acoustic emission (AE) waveforms in the rocks. Therefore, AE characteristic parameters, particularly *RA* and *AF* values, two AE indicators related to AE waveforms [9], are commonly utilized to identify rock micro-fracture modes. The acoustic signal of tensile damage in Fig 6(B) propagates predominantly as longitudinal waves with high *AF* values and low *RA* values. The acoustic signal of shear damage is mainly propagated as a shear wave with a high *RA* value and a low *AF* value. The middle-dashed line represents the transition between the two modes of fracture. *RA*-*AF* values have been gradually used to determine the type of fracture, but there is no uniform standard for the classification of tensile and shear fractures [22, 24].

Referring to the conventional diagonal method [57], the approximate 1:1 curve of the *RA*-*AF* maximum in this test is used as a transition line, which can still provide a good insight into the evolution patterns of the propagation in tensile or shear fracture events at different stages, even though the number of tensile and shear fractures cannot be quantitatively detected. The *RA* and *AF* values are calculated as follows:

$$RA=\frac{RT}{A}$$ (3)

$$AF=\frac{C}{D}$$ (4)

Where *RT* is the rise time in AE events, *A* is the maximum amplitude, *C* is the ring counts of one AE event, and *D* is the duration of one AE event.

**Fig 7:**
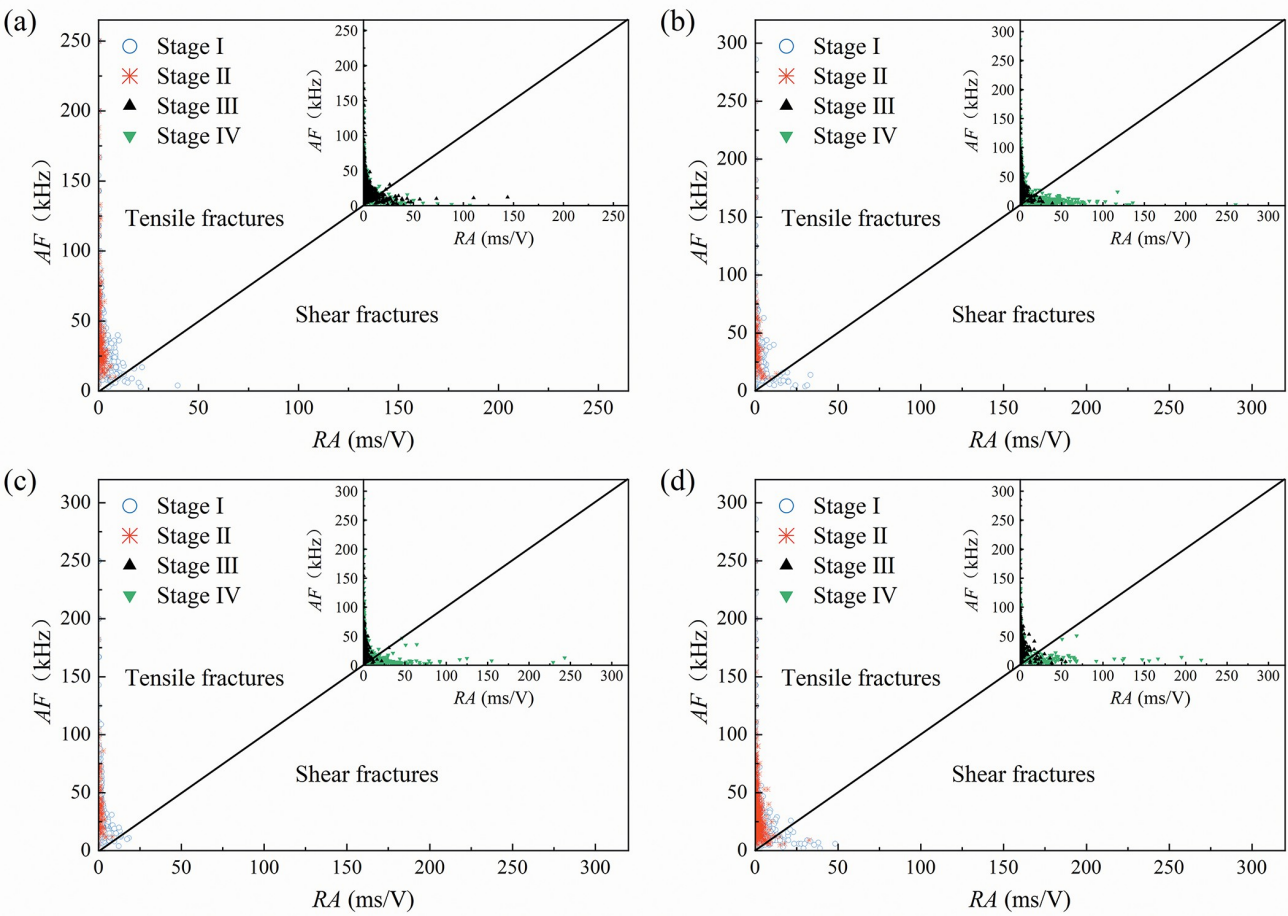
*RA* versus *AF* variation in the AE data related to fine yellow sandstone specimens (a) 0°specimen, (b) 30°specimen, (c) 60°specimen, (d) 90°specimen.

**Fig 8:**
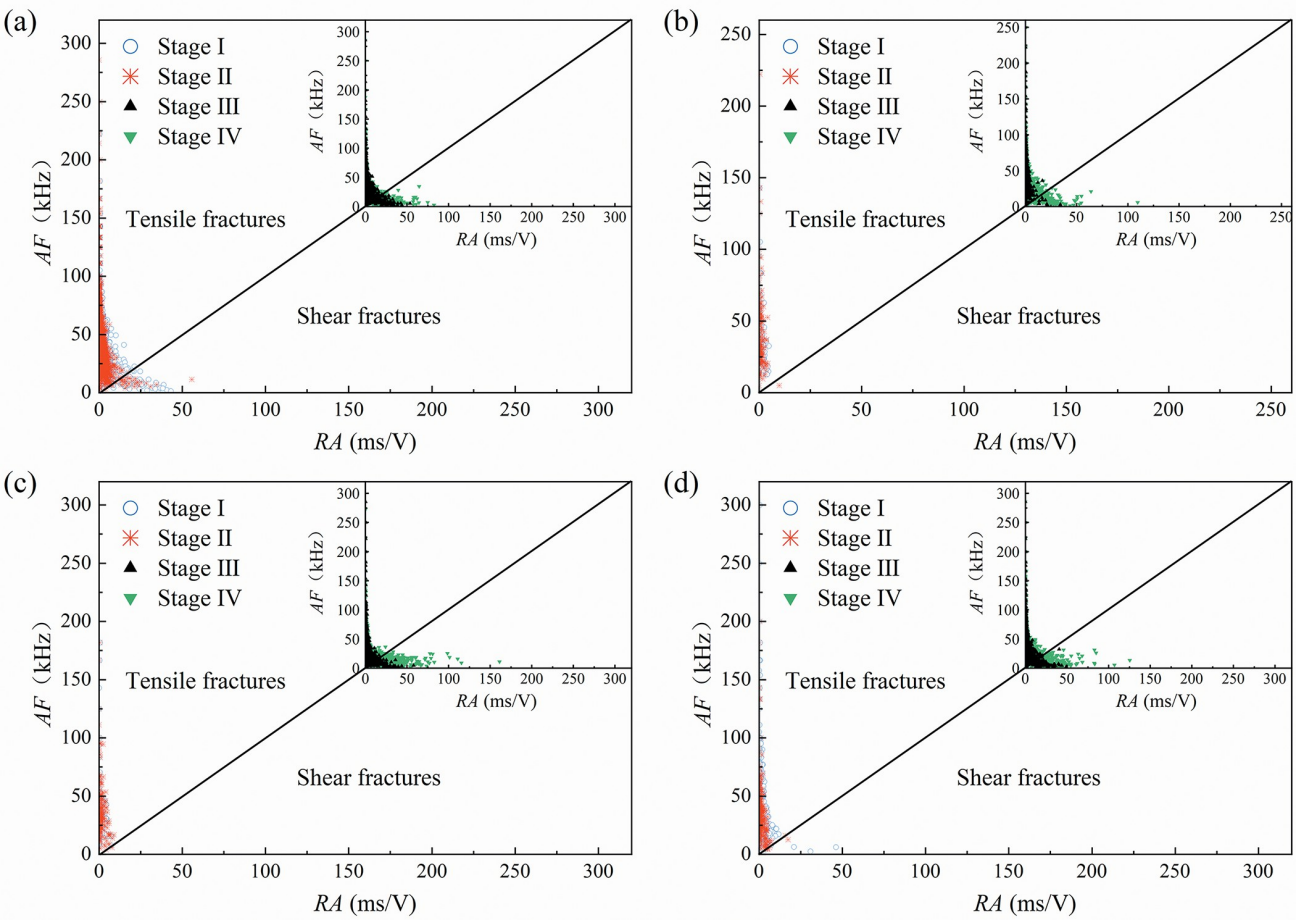
*RA* versus *AF* variation in the AE data related to red sandstone specimens (a) 0°specimen, (b) 30°specimen, (c) 60°specimen, (d) 90°specimen.

Figs 7 and 8 show the *RA*-*AF* variation of the sandstone specimens subjected to uniaxial compression tests. Take 0°, 30°, 60°, and 90° specimens as examples. For the fine yellow sandstone, the micro-fracture events of the 0° specimen in Fig 7(A) are predominantly tensile with a few shear fractures in stage I, while almost all of them are tensile in stage II. The *RA-AF* distribution is comparable in stage III and stage IV, while stage IV exhibits relatively more signals with high *RA* values and low *AF* values, i.e., more shear fractures. It demonstrates that the shear fracture within the specimen is mainly distributed in the damage process. The rock also shows a few shear fracture events during the compacting process, but nearly none during elastic deformation. In stages I and II, the *RA-AF* distribution of the 30° specimen in Fig 7(B) resembles that of the 0° specimen, but there are much more shear fracture events in stage IV than in stage III. During plastic deformation, the shear form dominates the micro-fracture events within the specimen. As can be seen in Fig 7(C) and Fig 7(D), the fraction of shear fracture events in stage IV increases as the dip angle increases. The pre-existing flaws have a negligible impact on the micro-fracture characteristics during the compacting and elastic deformation stages. The main effect is in stage IV, which increases the possibility of shear fracture events.

For the red sandstone, the 0° specimen in Fig 8(A) displays numerous micro-fracture events in the form of tension and shear in stages I and II, with *RA* values densely distributed between 0 and 45 ms/V. The shear fracture events in Stage III and Stage IV are comparable to those in the fine yellow sandstone, with a higher proportion of shear fracture events and a more concentrated distribution of *RA* values in the higher interval. The 30° specimens (Fig 8(B)), 60° specimens (Fig 8(C)), and 90° specimens (Fig 8(D)) also have fewer shear fracture events in stages I and II, and more shear fracture events in stages III and IV, with the greatest number of shear fracture events occurring in stage IV.

It can be concluded that the *RA-AF* distribution characteristics are closely related to the shape of the pre-existing flaws. The *RA* and *AF* values represent, respectively, the amplitude of shear and tensile waves. During the plastic deformation stage, the dense distribution interval of *RA* values for the two sandstone types indicates an increase in internal shear micro-fracture events during crack initiation. The amplitude of shear waves in stages III and IV increases with the increase of the inclination of the flaws, and the proportion of shear fracture events increases as well.

### 3.3 Surface crack evolution

**Fig 9:**
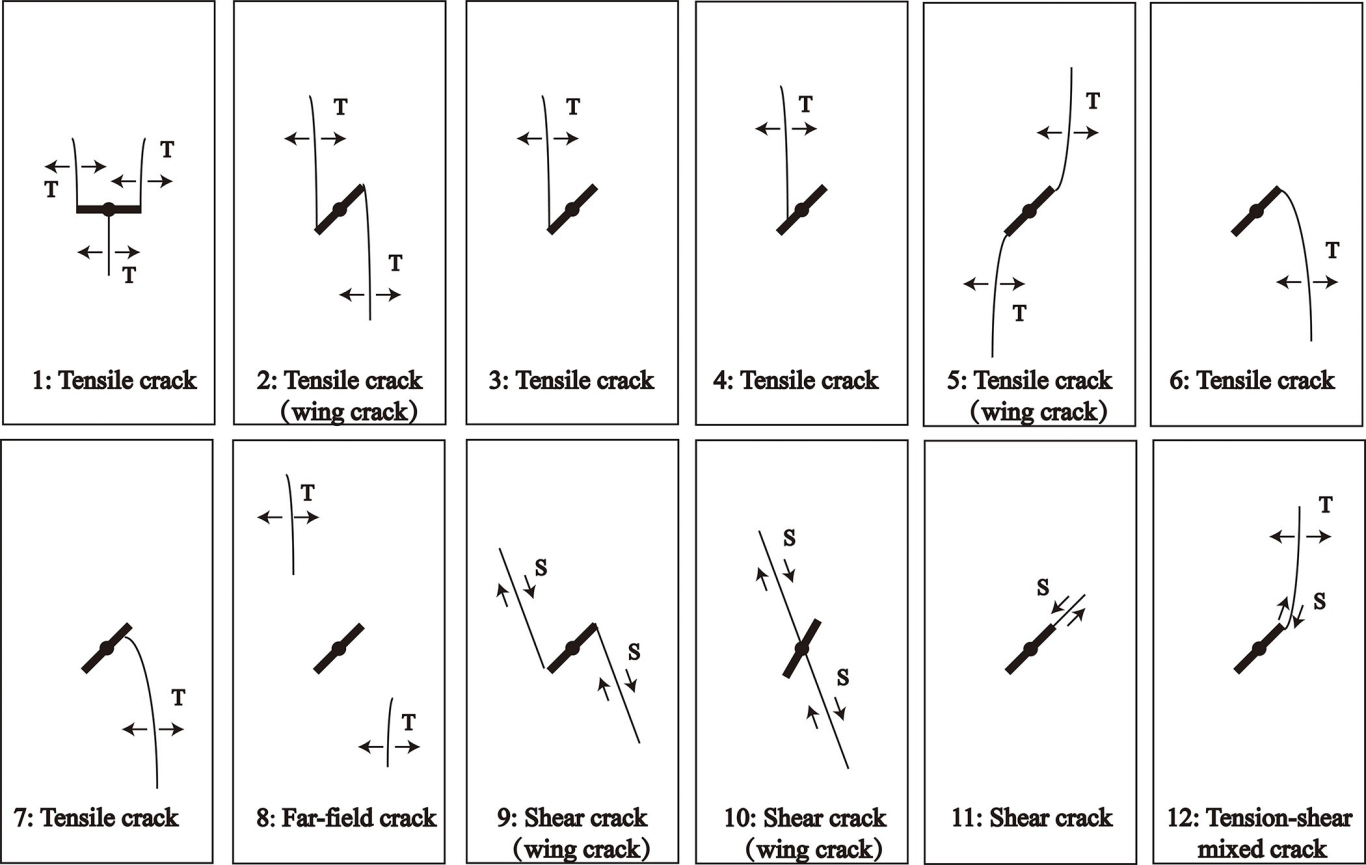
Types of crack propagation counted in the present study. T tensile crack, S shear crack [3].

Surface cracking usually occurs in the final stages of micro-fracture or separation within a rock. Two industrial cameras recorded the uniaxial loading process of all specimens. Twelve crack types (Fig 9) were identified based on crack-growth trajectories and crack initiation mechanisms [3]. Among them, tensile cracks can be subdivided into seven types, which are crack types 1 to 7. Shear cracks are subdivided into three types, which are crack types 9 to 11. In addition, there are far-field crack type 8 and tensile-shear mixed crack type 12. The specific characteristics of the above-mentioned crack types are as follows.

1. Crack type 1: The crack is a special type of crack in this study due to the presence of the pre-digging hole in the middle of the specimen. Two tension cracks in the axial stress direction appear at both ends of the flaw, while a tension crack propagating in the opposite direction appears in the central pre-digging hole. The three cracks appeared almost simultaneously.
2. Crack type 2: tensile cracks are initiated at both ends of the flaw and form wing cracks roughly parallel to the axial stress direction.
3. Crack type 3: tensile cracks initiate at the tip of the flaw, and usually the crack propagation is parallel to the axial stress direction.
4. Crack type 4: tensile crack initiates at the flaw (non-tip), usually the crack propagation direction is parallel to the axial stress direction.
5. Crack Type 5: Two tensile wing cracks initiated simultaneously at both ends of the crack grow along the direction parallel to the flaw in the early stage and gradually deviate in the direction of axial stress.
6. Crack type 6: tensile crack initiated at the tip of the flaw. The crack grows along the vertical flaw direction at the beginning and gradually deviates to the axial stress direction at the later stage.
7. Crack type 7: tensile crack initiated in the flaw (non-tip). The crack grows along the vertical defect direction at the beginning and gradually deviates to the axial stress direction at the later stage.
8. Crack type 8: The location of the far-field crack initiation is not near the pre-existing flaw. In this study, this type of crack propagation direction is mostly parallel to the axial stress direction, which is a kind of tensile crack.
9. Crack type 9: Shear wing crack initiated at the tip of the flaw. The two cracks are parallel to each other, and the path is smooth.
10. Crack type 10: Shear wing crack initiated in the pre-digging hole, and the growth directions of the two cracks overlap each other.
11. Crack type 11: Shear crack initiated at one of the tips of the flaw. The crack propagation path is parallel to the pre-existing flaw and the displacement direction is vertical to the flaw.
12. Crack type 12: tensile-shear mixed crack initiated at the tip of the flaw. The crack grows in the direction parallel to the flaw in the early stage as a shear crack. At the later stage, it gradually deviates in the direction of axial stress and is a tensile crack. Crack type 12 is similar in appearance to crack type 5. However, there is a distinct difference in the actual propagation mechanism of the two types of cracks. It can be distinguished by the direction of displacement of the cracks as seen in the recorded video.

**Fig 10:**
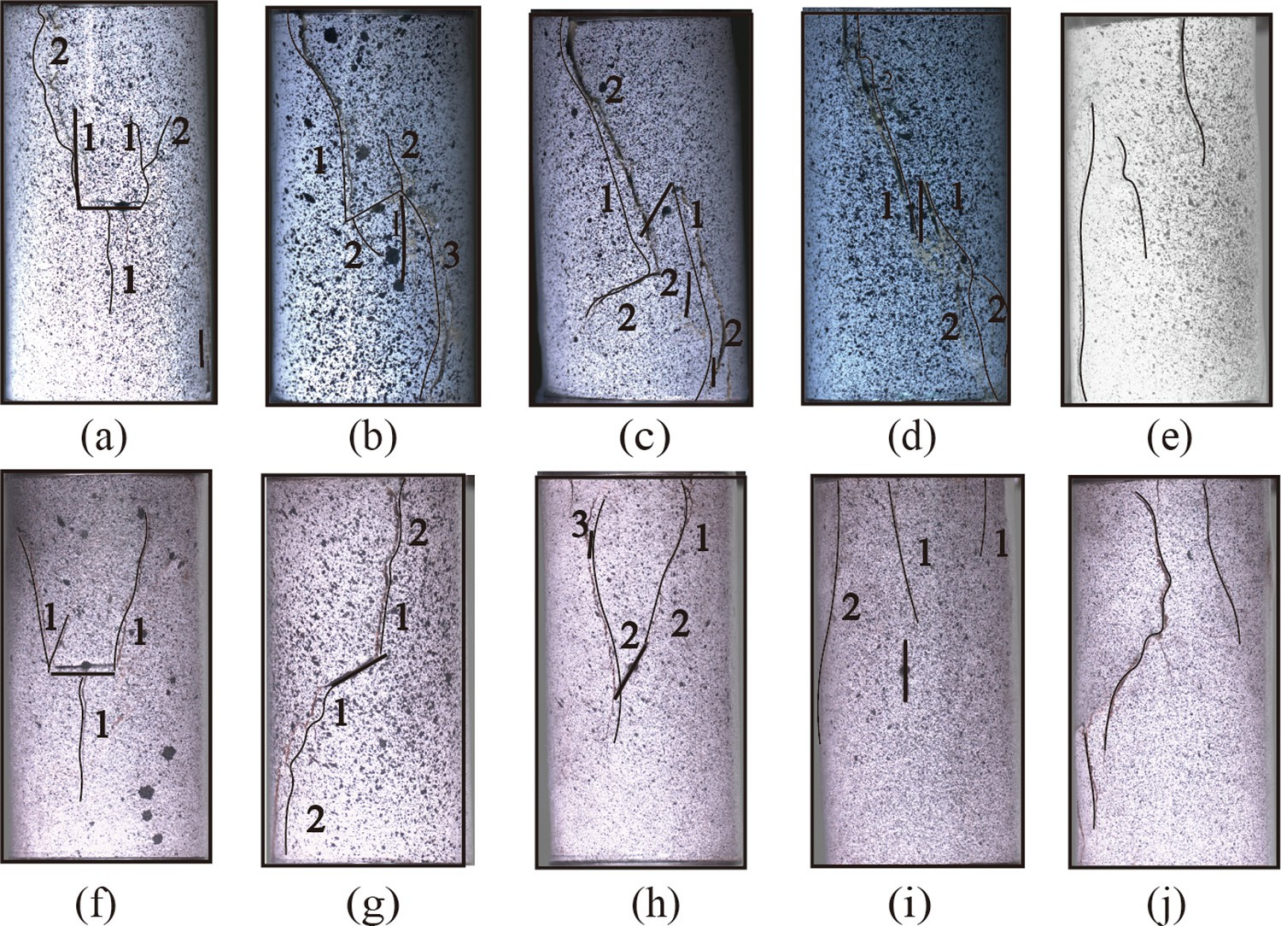
Ultimate failure modes and crack distribution characteristics of sandstone specimens (a-e) 0°, 30°, 60°, 90°and intact fine yellow sandstone specimens, (f-j) 0°, 30°, 60°, 90°and intact red sandstone specimens.

Fig 10 depicts the final failure mode and crack distribution characteristics of many specimens, with the figures sorted in the order of crack initiation. For the fine yellow sandstone, the 0° specimen (Fig 10A) first formed the typical type 1 crack, followed by secondary crack penetration causing specimen failure. The 30° specimen (Fig 10B) exhibited a type 2 wing crack, followed by a type 7 crack on the lower left side of the flaw and a type 6 crack on the upper right side. The final failure of the specimen was still triggered by the wing crack penetration. The final failure of the 60° and 90° specimens (Fig 10C and 10D) was caused by the type 9 shear wing crack formed on the upper left and lower right sides of the flaws. The red sandstone has a far lower number of cracks than that of the fine yellow sandstone, which is consistent with the results of acoustic emission tests. Notably, the crack initiation in the 60° and 90° specimens (Fig 10C and, 10D) did not originate from the pre-existing flaws. Typically, tension cracks were initiated at the top of the specimens, which occurs in intact specimens. Specimen damage, particularly in 90° specimens, is a process of progressive fracture of longitudinal tension cracks, not instantaneous damage.

Table 3 summarizes the types of cracks that have been observed in all specimens, with the size of the numbers in the table representing the times that one type of crack has occurred. Type 1 crack is mainly found in flawed specimens with a low dip angle and is the primary cause of damage to 0° and 15° specimens. Type 2 and 3 cracks are the most common tensile cracks, and shear cracks and far-field cracks are mainly distributed in specimens with a dip angle greater than 45°. Crack type 9 is the most common shear damage in flawed specimens with a great inclination, and the crack formation time is quite short. As the inclination of the flaws increases, the shear crack controls the failure of the specimen, resulting in a more brittle specimen. However, the influence of pre-existing flaws on crack propagation is diminishing. When the initial crack no longer initiates in the pre-existing flaws but instead forms a longitudinal far-field crack leading to specimen failure, the brittleness of the specimen weakens once more.

**Table 3:** Crack types of sandstone specimens containing single flaws with different angles.

Specimens	Angle (°)	Crack Types											
		1	2	3	4	5	6	7	8	9	10	11	12
fine yellow sandstone	0°	3		3									
	15°	2	2	1	1			1	1				
	30°	7	2	2			3	1					
	45°		3	2					2			2	1
	60°		1						2	2		1	
	75°		1	3					2	2			
	90°								3	1	2		
red sandstone	0°	2	1	1									
	30°		1		1	2	1		1				
	60°			3		1			2			2	
	90°								3				

## 4. Deformation and cracking mechanism

### 4.1 Cracking-induced evolution of the SLZs

**Fig 11:**
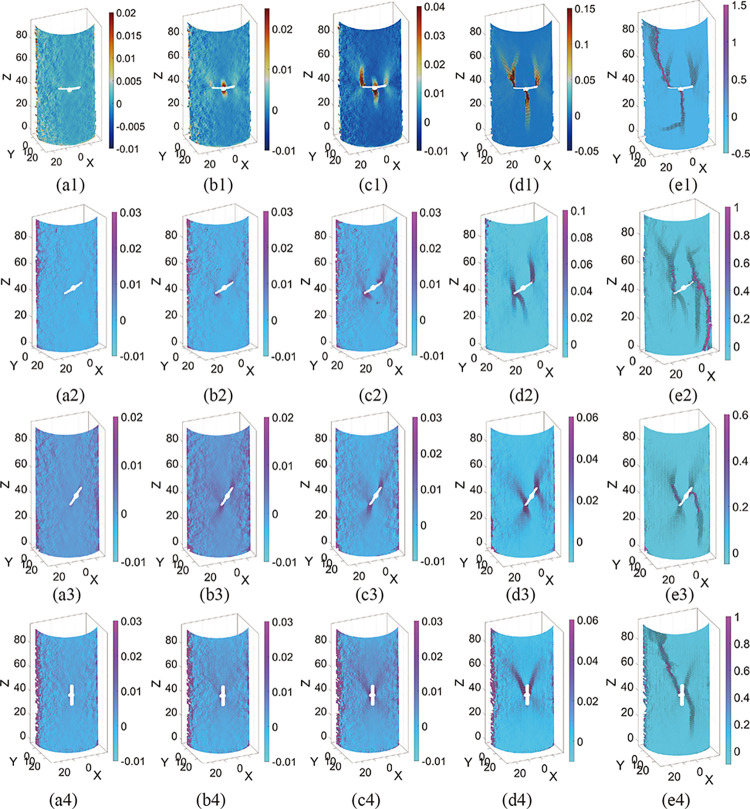
Evolution of the SLZs in fine yellow sandstone specimens, (a1-a4) refer to the 0° specimen, 30° specimen, 60° specimen, and 90° specimen, respectively, (a-e) refers to the stress at *σ*_*cc*_, *σ*_*ci*_, *σ*_*cd*_, *σ*_*p*,_ and failure, respectively.

**Fig 12:**
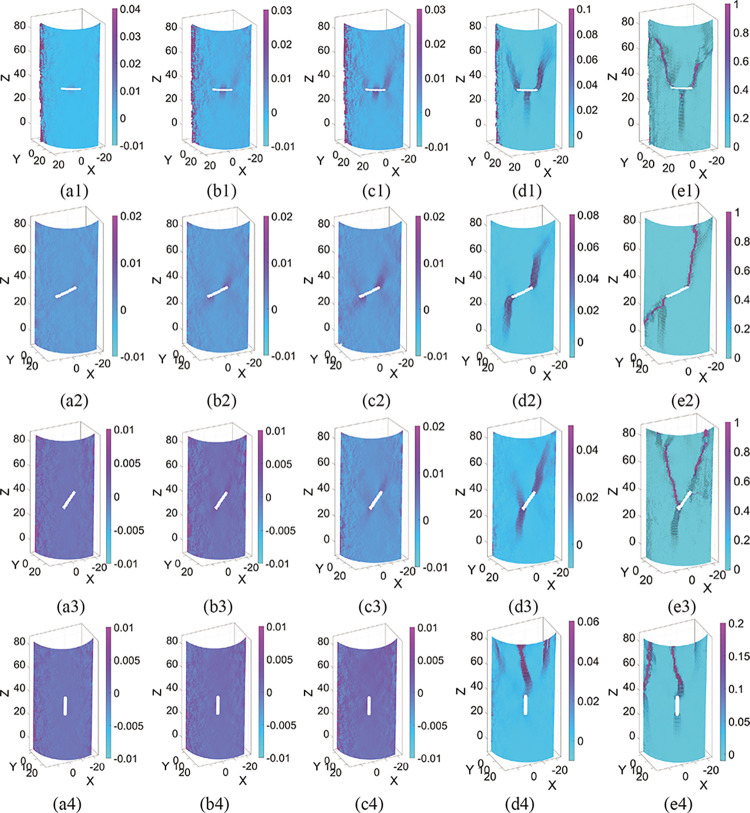
Evolution of the SLZs in red sandstone specimens, (a1-a4) refer to the 0° specimen, 30° specimen, 60° specimen, and 90° specimen, respectively, (a-e) refers to the stress at *σ*_*cc*_, *σ*_*ci*_, *σ*_*cd*_, *σ*_*p*,_ and failure, respectively.

Figs 11 and 12 depict the results of the 3D strain field evolution of the specimens solved by 3D-DIC. The time nodes of each image correspond to the typical stress values from Table 2. X, Y, and Z in the figure are the solved spatial coordinates of the specimen in mm. When the stress is *σ*_*cc*_, the strain field on the surface of all specimens is uniformly distributed. The specimens did not show strain localization zones (SLZs) under external forces [4], i.e., no localized extrusion deformation was produced. SLZs develop initially during the elastic deformation stage and have the same shape characteristics as the initial crack in the specimen, which can be used as a precursor to crack formation. When the stress is *σ*_*ci*_, SLZs with wing or anti-wing shapes appear near the tip of pre-existing flaws or pre-digging holes, and they tend to propagate outward. The range of SLZs decreases as the dip angle of the pre-existing flaws increases. Meanwhile, the micro-crack has been initiated and is in a stable growth state. When the stress is *σ*_*cd*_, the SLZs formed at the flaw are clear. The micro-cracks gather into nuclei, and macro-cracks emerge. Similarly, the scale of cracks is reduced in specimens having large inclination flaws. With the accelerated propagation of the crack, the crack aggregation penetrates at the peak stress *σ*_*p*_. The specimen is in a state of critical instability, resulting in complete failure. In Fig 12, the red sandstone is more difficult to form SLZs than the fine yellow sandstone in the same deformation stage.

Under uniaxial compression, pre-existing flaws are prone to causing stress concentration, particularly at their tips. Consequently, when the stress surpasses the local strength, microcracks initiate at these flaw tips. These microcracks nucleate and propagate in the direction of maximum shear stress, leading to the formation of wing cracks. The direction of maximum shear stress depends on the angle of the pre-existing flaws. When the dip angle of the flaw is small, the upper and lower parts of the flaw are prone to relative deformation. This is the main reason for the formation of the type 1 crack. As the dip angle increases, the stress concentration is transferred to the tip of the flaw, resulting in the easy formation of SLZs at the tip of the flaw. The propagation of SLZs at both ends of the flaw deviates toward the axial stress direction, increasing the likelihood of shear damage to the specimen. This is consistent with the law presented in Table 3. Additionally, deformation of the upper and lower portions of preexisting flaws is relatively challenging. It can explain why the brittle properties and strength of the sandstone increase with the increase of the dip angle of the flaws.

The surface crack propagation characteristics of the two types of sandstone specimens are partially similar, especially the crack propagation pattern and direction of the flawed specimens with the same dip angle. This cracking behavior can be attributed to flawed settings. Crack initiation and coalescence in rocks under load are largely controlled by pre-existing flaws, which also initiate a progressive damage process in the rock. In contrast, the magnitude and pattern of damage progression differs across the two types of sandstone specimens. Red sandstone specimens are more brittle, and crack formation and propagation are more difficult and rapid. When the dip angle increases, the SLZs are no longer initiated at the flawed edges. It corresponds to the previously described diminishing influence of pre-existing flaws on crack propagation.

### 4.2 Cracking-induced AE event characteristics

**Fig 13:**
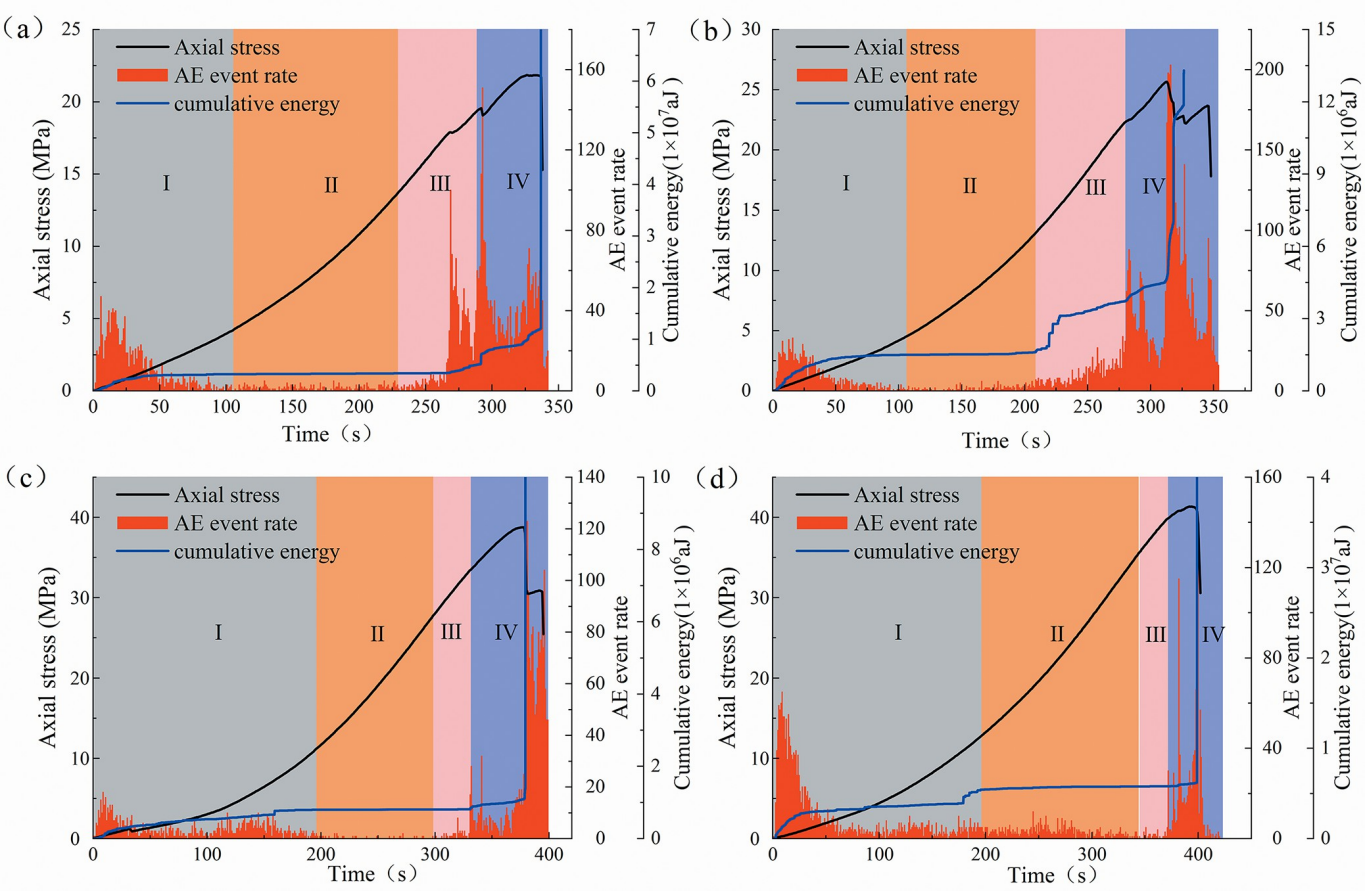
Evolution of AE event rate and cumulative energy of fine yellow sandstone specimens (a) 0°, (b) 30°, (c) 60°, (d) 90°.

**Fig 14:**
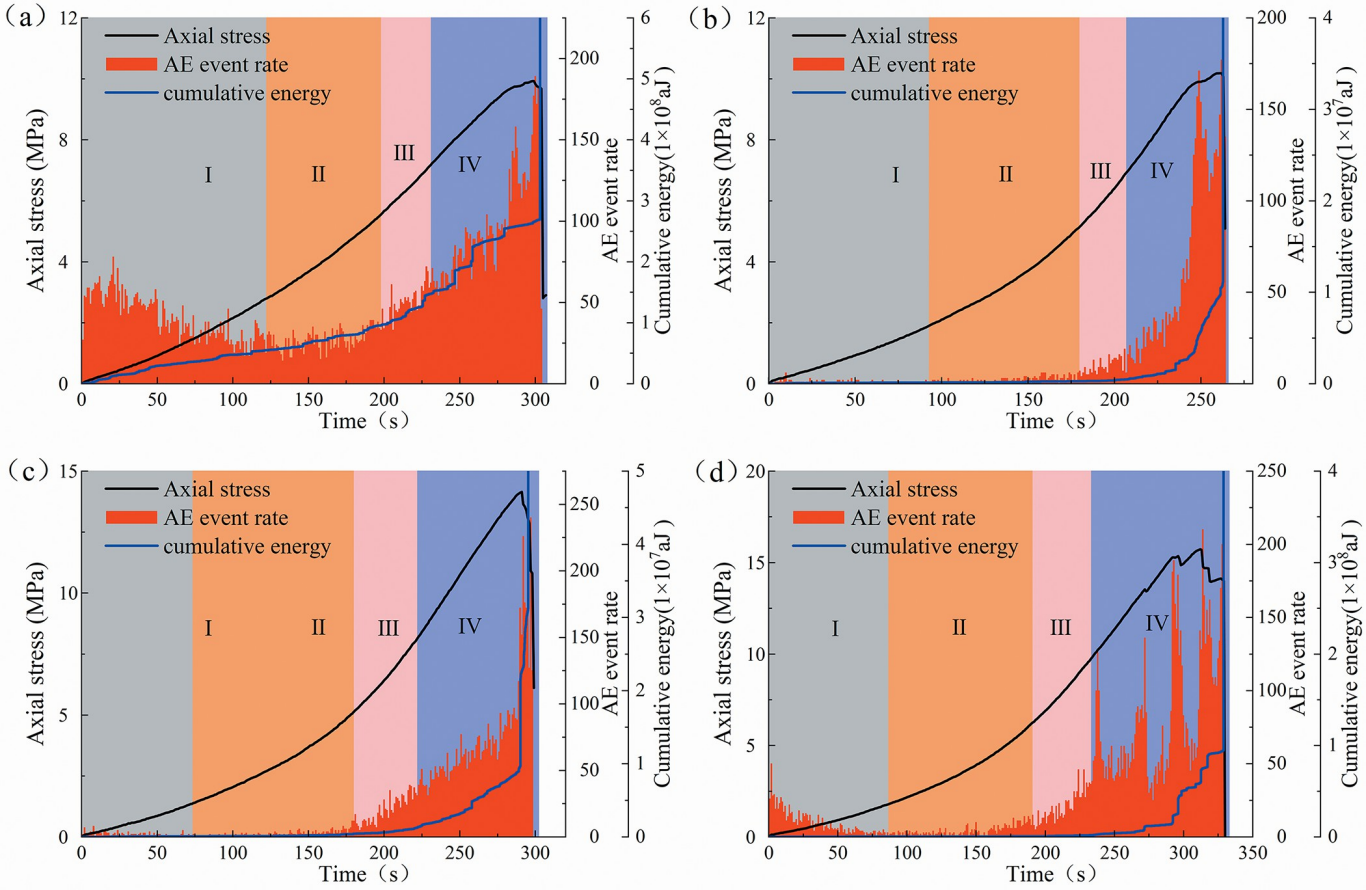
Evolution of AE event rate and cumulative energy of red sandstone specimens (a) 0°, (b) 30°, (c) 60°, (d) 90°.

Figs 13 and 14 display the evolution characteristics of AE event rate and cumulative energy with stress-time for the two types of sandstone specimens. The AE event rate is the number of AE hits per unit of time and is used to determine the frequency of damage deformation of the specimen. The accumulated energy is the time integral of the squared sensor signal voltage before amplification, expressed in aJ (ajoule), and is the intensity of the damage deformation.

It can be seen from Fig 13 that the fine yellow sandstone has a high AE event rate and energy release in stage I. This is due to the closure of micro-fissures within the rock under pressure and the contact friction between the bottom of the specimen and the pad, a large number of AE events are produced. Stage II can be considered as the " quiescent area ", which produces essentially no AE events or energy release. At Stages III and IV, the AE events are active and the rate of energy release remains a high rate until the specimen failure. Generally, the dip angle of the flaw affects the damage deformation behavior of rocks. In Fig 13(A), the 0° specimen undergoes several energy releases and AE events during Stage III, indicating a high-frequency, high-amplitude damage behavior. However, the inactivity of AE events before the peak stress increases with the increase of the dip angle (Fig 13C and 13D). Also, the appearance of stage III is lagging and of shorter duration. It is demonstrated that the specimens containing pre-existing flaws with low dip angles generate more local damage in the early stages of loading. Flawed specimens with a high dip angle generate less local damage in the pre-loading period, but the damage was more abrupt, i.e., more brittle, after the peak loading period.

The dip of the flaw has less effect on the brittleness of the red sandstone. This distinction stems from the different structural characteristics of the two types of sandstones, with the red sandstone being difficult to generate localized, small-scale fractures. As depicted in Fig 14, the frequency of AE events generated by the red sandstone grows gradually without a sudden energy release process. It indicates that its internal damage deformation is a continuous accumulation and development process. However, the 90° specimen exhibited local damage before the peak stress (Fig 14D). It is owing to the specimen damage induced by longitudinal tension fracture and crack propagation independent of the pre-existing flaws.

## 5. Discussion

**Fig 15:**
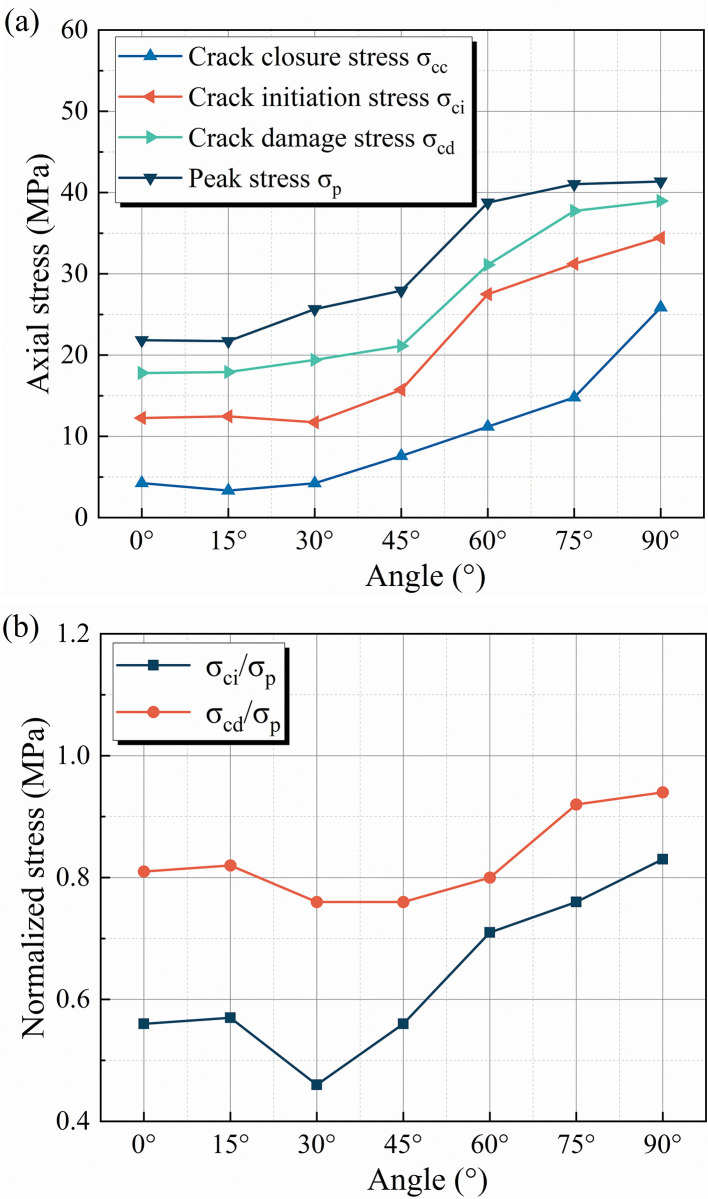
Cracking levels with regard to dip angle. a Crack initiation and damage stresses. b Normalized crack initiation and damage stresses [58].

The dip of the initial flaw and the variation in rock material are the two most influential factors in the cracking behavior of brittle rocks. The axial load on the inclined flaw can be decomposed into normal and tangential stresses. As the inclination of the faults rises, the tangential stress at both ends of the flaw increases, resulting in shear damage to the specimen. In addition, the dip angle of the flaws affects the macro-deformation behavior of the sandstone. The crack initiation and irreversible damage of the specimen depicted in Fig 15 require greater stress to be guided [58], resulting in a more brittle specimen. The material variation is mainly in the accumulation mode of micro-fracture events. In this investigation, the damage process within the red sandstone showed gradual accumulation, while the fine yellow sandstone had multiple AE energy releases after crack initiation. This is the direct reason for the difficulty in initiating and rapid propagation of cracks in red sandstone. Furthermore, the pre-existing flaws in red sandstone have a significantly weaker effect on crack propagation. When the dip angle of the pre-existing flaws is greater than 60°, the crack no longer initiates at the flaws, but rather at the bottom or top position of the specimen, much as it does when the intact specimen is damaged. Presumably due to the stronger brittle properties of red sandstone, the stresses in flawed specimens with large dips are no longer concentrated at the edges of the flaws. It is also evident from Fig 12 (d4) that the SLZs appear initially near the top of the specimen and not at the edge of the flaw.

Typically, cracking behavior is accompanied by corresponding strong acoustic-optical evidence. In this study, the internal micro-fracture modes and surface cracks of the specimens were distinguished based on the continuously observed acoustic-optical data. Table 4 displays the main distribution ranges of *RA* and *AF* values of fine yellow sandstone specimens at various deformation stages. *RA* and *AF* values represent the amplitude of shear and tensile waves, respectively, and the shear fracture rate is the proportion of shear events in micro-fracture events within the rock. The main distribution range of *RA* values is greatest during the crack unstable propagation stage (IV), followed by the crack stable propagation stage (III), the crack closure stage (I), and the smallest in the elastic deformation stage (II). The amplitude of the shear wave increases with the macro-deformation scale while causing more shear fracture events within the specimen. The main distribution range of *RA* values increases from 0–50 ms/V to 0–219 ms/V, and the shear fracture rate increases from 10.74% to 22.12%, as the dip angle of the pre-existing flaws increases. The above results demonstrate that the number and scale of shear micro-fracture events within the rock are increasing.

**Table 4:** The main distribution range of *RA* and *AF* values during the cracking process of fine yellow sandstone specimens.

Angle (°)	Macro-deformation stage	*RA* (ms·V^-1^)	*AF* (kHz)	Shear fracture rate (%)
0°	I	0–25	0–100	1.41
	II	0–9	0–150	0
	III	0–50	0–150	4.88
	IV	0–50	0–150	4.45
30°	I	0–34	0–100	1.83
	II	0–13	0–100	0
	III	0–40	0–200	1.43
	IV	0–140	0–150	10.12
60°	I	0–19	0–110	0.94
	II	0–9	0–110	0
	III	0–31	0–200	2.53
	IV	0–100	0–200	11.34
90°	I	0–49	0–150	2.76
	II	0–33	0–150	1.21
	III	0–56	0–150	4.99
	IV	0–219	0–150	13.16

**Fig 16:**
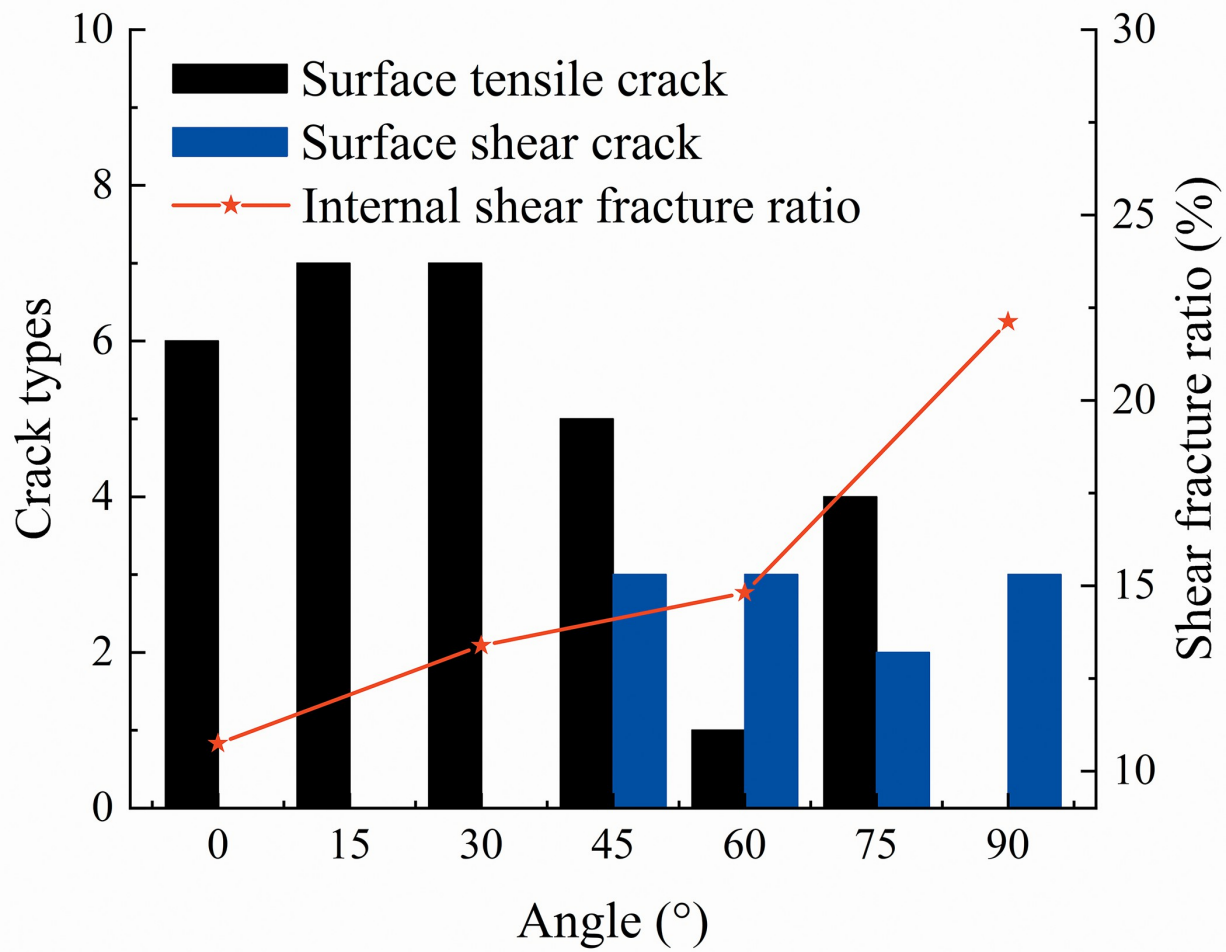
The correlation between shear fracture events and surface cracks at different dip angles.

The correlation between internal micro-fracture modes and surface crack types is compared in Fig 16. For clarity, in this paper, ’tensile-shear cracks’ denote cracks visible on the surface (refer to Table 3), whereas ’tensile-shear damage modes’ describe the internal micro-fracture modes within the specimen that are invisible. In the 0°, 15°, and 30° specimens, for instance, there are no surface shear cracks, but the proportion of shear fracture events within the specimens is growing. But there is also some potential relationship between these two cracking behaviors; the shear fracture rate increases with the dip angle of the flaw, and shear cracks also tend to appear in flawed specimens with large dips. To a certain extent, the coalescence behavior of rock surface cracks is a macroscopic characterization of internal micro-fracture events, and the higher the shear micro-fracture rate, the more likely to initiate shear cracks.

The evolution of SLZs is an optically compelling indicator of the type of surface cracks. The location and propagation direction of the SLZs vary with the brittle and tensile-shear characteristics of the specimens. The concept of SLZs is the white patches associated with microcrack development found by Wong and Einstein [32, 59]. The white patches exist in pre-existing flaws in the marble before the surface crack initiation. Previous research has identified white patches as an indicator of microcrack nucleation [44], which occurs during the elastic deformation stage. SLZs also appear before the initiation of surface cracks and are predictors of the incipient microcracks. Since the measurement of DIC requires the spraying of black speckles on the surface of the specimen, the white patches are difficult to notice and the time of appearance of SLZs is difficult to identify accurately. Therefore, it is challenging to determine the exact correlation between the two. In compression testing, a noteworthy difference between the fine yellow sandstone and the red sandstone is that the SLZs generated in the fine yellow sandstone are more distinct.

This study analyzes the acoustic-optical-mechanical characteristics associated with the failure process of flawed rocks and comprehensively evaluates their cracking behavior. It is well known that the inclination angle of the flaw affects the brittleness and cracking mechanisms of rock materials [3, 4, 10]. We utilized acoustic-optical data to reveal and explain the formation mechanism of this phenomenon. It was confirmed that different cracking behaviors are accompanied by corresponding strong acoustic-optical evidence. The findings of this study can be used to predict the cracking and collapse behavior of brittle rock masses in rock slope engineering.

## 6. Conclusions

1. In the crack initiation stage, many tensile fractures are initiated and coalesced with the occurrence of SLZs on the specimen surface. In the propagation stage, the micro-fractures coalesce into micro-fractures that propagate in tensile mode. Finally, the penetrating cracks break in tensile mode or slide against each other in shear mode.
2. Acoustic and optical perspectives are used to reveal how flaws and materials affect the strength and cracking mechanisms of brittle rocks. As the dip angle increases, the flaw tips are more likely to form SLZs, making it difficult for the upper and lower surfaces to deform relative to each other. This explains why the strength characteristics of the rock increase with the inclination angle. Additionally, the expansion direction of the SLZs at both ends of the flaw deviates from the direction of axial stress, increasing the likelihood of shear failure in the rock. The material influence is mainly manifested in the cumulative modes of micro-fracture events within the rock.
3. As macroscopic characterizations of micro-fracture events within the rock, surface cracks are difficult to observe but can be predicted by SLZs during the elastic deformation stage. The dip angle of pre-existing flaws modifies the location and propagation direction of SLZs, hence influencing the tensile-shear mode of the surface cracks.
4. Cracking behavior is accompanied by corresponding strong acoustic-optical-mechanical evidence. AE event rates and AE signals with high RA values increase during the crack propagation stages. The more brittle the specimen is, the more difficult the SLZs are to form. The higher the internal micro-shear fracture event rate, the more likely surface shear cracks are formed.

Looking ahead, since 3D-DIC is used for three-dimensional crack evolution monitoring, spatial SLZs can be employed to identify tensile-shear crack patterns. This approach can be cross-referenced with the AE method for a more comprehensive analysis.
